# Uptake of human papilloma virus vaccination among adolescent girls living with HIV in Uganda: A mixed methods study

**DOI:** 10.1371/journal.pone.0300155

**Published:** 2024-08-08

**Authors:** Violet Nakibuuka, Martin Muddu, Jean Pierre Kraehenbuhl, Caroline Birungi, Fred C. Semitala, Andrew K. Tusubira

**Affiliations:** 1 Makerere University Joint AIDS Program (MJAP), Kampala, Uganda; 2 Makerere University School of Medicine, Kampala, Uganda; 3 University of Lausanne, Vaud, Switzerland; 4 Department of Medicine, Makerere University College of Health Sciences, Kampala, Uganda; 5 Department of Community Health and Behavioural Sciences, School of Public Health, College of Health Sciences, Makerere University, Kampala, Uganda; 6 Faculty of Public Health and Policy, Department of Global Health and Development, London School of Hygiene and Tropical Medicine, London, United Kingdom; 7 MRC/ UVRI and LSHTM Uganda Research Unit, Entebbe, Wakiso, Uganda; Makere University College of Health Sciences, UGANDA

## Abstract

**Background:**

Human Papilloma Virus (HPV) vaccination can prevent more than 90% of cancers caused by HPV. Although this vaccination is recommended and provided at no cost to all adolescent girls aged 9 to19 years in Uganda, its uptake remains low. We sought to determine the uptake of, and factors associated with HPV vaccination among adolescent girls living with HIV in Uganda.

**Methods:**

We conducted an explanatory sequential mixed methods study, among adolescent girls living with HIV, attending HIV care at the Mulago ISS HIV clinic in Kampala, Uganda. We administered a structured questionnaire to elicit data on HPV vaccination and its covariates to a systematic random sample of 264 adolescent girls with HIV. A participant who had received all the three recommended HPV vaccine doses was classified as fully vaccinated. We then conducted four focus group discussions among adolescent girls living with HIV (n = 32), eight in-depth interviews among their parents and five Key informant interviews among their healthcare providers. We conducted descriptive statistics and logistic regression analyses for the quantitative data before thematic analysis for the qualitative data.

**Result:**

Of 264 adolescent girls, 31% (83/264) had at least one HPV vaccine dose; 22% (59/264) two doses, while 8.0% (21/264) were fully vaccinated (received three doses). While most participants received their first and second doses (48% (40/83)) and 57.6% (34/59), respectively) from school, the largest number of participants (47.1% (12/21)) received their third dose at community outreaches. Participants who received counseling from community members were three times more likely to get fully vaccinated compared to those who did not receive counseling (aOR 3.28, Cl:1.07–10.08, P = 0.038). From the qualitative follow-up, three major themes were identified: (1): Limited information about HPV vaccination, which gave room for misconceptions and doubts about the vaccine; (2) Parental influence on adolescent decisions was strong despite parents having limited knowledge about HPV vaccination and (3) Inadequacy of HPV vaccination services at the hospital and in the schools.

**Conclusion:**

Full HPV vaccination was low among adolescent girls living with HIV. Counseling of the adolescents by community members, alongside HPV vaccination community outreaches, provided a platform for vaccination. There should be strategies to provide adequate information about HPV vaccine to health workers, parents, and the adolescents. In addition to schools, community-based initiatives, including outreaches and lay-health workers can be utilized to improve HPV vaccine uptake among girls with HIV.

## Introduction

Globally, the prevalence of Human Papilloma Virus (HPV) infections is disproportionately higher in Low and middle-income countries and highest in the Sub-Saharan region at an estimated prevalence of 24% [1,2]. Moreover, HPV prevalence is higher among women living with HIV in the Sub-Saharan region, reaching estimated levels of 80% in Zambia and above 90% in Uganda [1]. Being infected with high-risk, cancer-associated, types (16 and 18) of HPV has been associated with the development of cancer of the cervix and other anogenital cancers [3,4]. Approximately, 70% of all cases of cancer of the cervix are caused by HPV-16 and 18 [5]. During the era of combination Antiretroviral Therapy (cART), the incidence of HPV-associated cancers and poorer outcomes remains elevated in Persons Living with HIV (PLHIV) relative to the general population [6]. There is therefore an urgent need to strengthen known cost-effective measures that can mitigate the incidence of HPV-associated cancers, particularly cancer of the cervix.

HPV vaccination could prevent more than 90% of cancers caused by HPV [7]. The World Health Organization (WHO) recommends HPV vaccines for all adolescent girls aged 9 to 19 years, including those living with HIV [8–10]. The Uganda Ministry of Health (MoH) introduced the Quadrivalent HPV vaccine that targets HPV types 6, 11, 16, and 18 to be used in the nationwide scale-up of immunization against cancer of the cervix [7]. The MOH implements HPV vaccinations for girls at health facilities, particularly hospitals, through routine immunization sites and in community outreaches. The MoH also organizes country-wide mass HPV-vaccination campaigns within schools.

Despite HPV vaccination being at no cost to recipients, its uptake has remained low in Uganda with approximately 14–30% of the eligible adolescents getting their first dose and less than 50% of targeted girls receiving their 2nd follow up dose [1,9,11–14]. A study conducted by Lydia et al at Mulago national referral hospital two years after the national rollout of HPV vaccination Programme in Uganda found a completion level of the second dose of HPV vaccine within 6 months of the first dose at 43.3% [12]. The WHO recommends a 2-dose schedule for immunization of immune-competent girls aged 9–14 years [12]. As per the UNICEF website by 2022, the HPV vaccine had been fully introduced in 130 countries. However, only 14 per cent of girls are currently fully protected against HPV, indicating the need for increased efforts in this area to scale up HPV vaccination [15].

Additionally, HPV vaccination coverage as well as associated factors among adolescent girls living with HIV are not well understood in Uganda. In this study, we determined the prevalence and associated factors of HPV vaccination among adolescent girls living with HIV at Uganda’s largest HIV clinic. Understanding the prevalence of HPV vaccination, associated factors and explanations of variations in uptake of HPV vaccine among adolescent girls living with HIV will lay the foundation for developing strategies to scale up the HPV vaccination intervention to adolescent girls who are at a higher risk for cancer of the cervix.

### Study objective

To determine the uptake and associated factors of Human Papilloma virus vaccination among adolescent girls living with HIV in Uganda.

## Methods

### Study design

We conducted an explanatory sequential mixed methods study between July and September 2021. We used a structured questionnaire to obtain quantitative data on HPV vaccination among adolescent girls living with HIV. This was followed with four focused group discussion (FGD) (n = 32), five key informant interviews (KII) and eight in-depth interviews (IDI) to obtain explanations for the HPV vaccination prevalence identified among the adolescent girls living with HIV.

### Study setting

This study was conducted at Mulago ISS, the largest HIV clinic in Uganda which provides comprehensive HIV services to over 16,500 PLHIV of all ages. Five percent of the patients receiving care at this clinic are aged less than 20 years. The clinic is located within Mulago National Referral and Teaching Hospital in Kampala, Uganda’s Capital City. HIV related clinical activities include HIV Testing and Counseling (HTC), provision of ART, screening for other HIV comorbidities and long term follow up. Mulago ISS Clinic is owned and operated by the Makerere University Joint AIDS Program (MJAP). Clinic services are provided by Doctors, Nurses, Clinical Officers, HIV Counselors, Laboratory Technicians, Pharmacy Technicians, and Records Officers with the assistance of community-based service providers who include PLHIV expert clients, adolescent peer supporters and lay workers. Mulago ISS Clinic does not offer HPV vaccination services. However, PLHIV who are eligible for HPV vaccination are referred to Mulago National Referral Hospital’s ward 15 which is the adolescent friendly clinic offering reproductive health services and various immunization programs for adolescents. Mulago National Referral Hospital ward 15 uses the Center for Disease Control and Prevention (CDC) guideline, which recommends HPV vaccination for girls including those aged 9 to 19 years.

Adolescents have a special space in the HIV clinic called the adolescent friendly corner in which services for only adolescents and young adults aged 10–24 years are provided. Adolescent and Youth-Friendly Health Services (AYFHS) are designed to address the barriers faced by youth in accessing high-quality services [23]. WHO defined characteristics of adolescent friendly health services as equitable, accessible, acceptable, appropriate and effective [24].

### Study population

Our participants for the quantitative study were adolescent girls living with HIV, aged 9–19 years, seeking care at Mulago ISS Clinic, Kampala who consented and accented to participate. We excluded adolescents who had stage three and/or four HIV disease because they are usually very sick, weak, and frail and spend very minimal time at the health facility.

The follow-up qualitative study included three populations: 1) adolescent girls living with HIV who participated in FGD; 2) Parents to the adolescent girls (IDI participants) and 3) Healthcare providers who participated as key informants.

### Sample size and sampling of quantitative participants

The quantitative study sample size was 264, estimated using the Leslie Kish formula $\;n=\frac{z^{2}pq}{\delta^{2}}$ [16] under the following parameters: 1.96 standard normal deviation(z); 22% estimated prevalence (p) of HPV vaccination in Uganda [11], a 5% level of precision(δ).

The qualitative sample size was determined following the maximum variation sampling strategy. Utilizing the maximum variation sampling strategy, adolescents were divided into two groups: those aged 9–15 years and 16–19 years. Two focus group discussions were conducted for each category and each group consisted of at least five to seven participants. Therefore, a minimum of four focus group discussions were conducted. For the key informant interviews, five health care providers who work in the adolescent friendly corner were interviewed. These included one doctor, one nurse, one counselor and two peer educators. We also conducted eight in-depth interviews with parents /guardians of the adolescents. We had two categories of parents/ guardians: parents living with HIV and parents not living with HIV. For each category, four parents /or guardians were selected as we considered two males and two females.

### Ethical considerations

We obtained Ethical approval to conduct the study from The AIDS Support Organization (TASO) Research and Ethics Committee TASOREC/045/2021-UG-REC-009 and Uganda National Council for Science and Technology under a registration number HS1436ES. We sought administrative permission from Mulago ISS Clinic. Both consent and assent were obtained from the guardians and the participants aged 09–17 years respectively while those aged 18–19 years consented for themselves.

### Sampling procedure for the quantitative study

We used systematic random sampling technique to reach the study participants. Our sampling frame was developed from the clinic database of adolescent girls aged 9–19 years living with HIV active in care at Mulago ISS Clinic at the time of data collection. A total of 16,500 clients were active in care in the clinic at the time of the study of which 398 were eligible. Sampling interval of 2 was arrived at by dividing the total number of eligible participants, 398 on the sampling frame by the target sample size (264).

Since the adolescent clinic ran every Wednesday, adolescents arrived at the clinic and were given numbers according to their arrival, from 1 counting to the last attendant of the clinic. We then made a random start for the initial female adolescent and thereafter followed the interval of every second female adolescent coming into the clinic. This selection would continue until the last client came in. After selecting an adolescent, the interviewer would approach her and explain the purpose of the study, screened her for eligibility and requested her to consent. Only eligible adolescent girls who provided written informed consent were invited to participate in the study. All questionnaires were administered after routine healthcare, in order not to interrupt service delivery for the adolescent girls.

### Sampling and sample size of the qualitative participants

We purposively selected all the qualitative participants using the maximum variation sampling strategy, which enabled us to obtain perspectives of different categories of our participants. This sampling strategy also informed the sample size for each qualitative data collection method. For the FGD, adolescents varied by age and were categorized into two: two FGD with adolescents aged 9–15 years and two groups with those 16–19 years. Each group had a maximum of eight participants.

For the Key informants, we purposively selected healthcare providers who worked directly with the adolescents (those that worked in the adolescent friendly corner) at Mulago ISS clinic. Health care providers who worked in the adolescent friendly corner included medical doctor, nurses, counselors and adolescent peer support. Following the maximum variation strategy, we selected at least one informant from each group hence a total of five key informant interviews. We also purposively selected eight parents to the adolescent girls living with HIV to participate in the in-depth interviews. To obtain a maximum variation, we considered HIV status of parents and gender. We selected four parents who were living with HIV and four who were not living with HIV (thus a total of eight participants). For each category of parents, two were male parents and two were females.

### Data collection procedures

Five research assistants with a nursing background were trained and collected the data under the supervision of the first author. These research assistants were not part of the staff at the health facility. We used a pretested structured questionnaires adapted from previous studies on HPV vaccine uptake in Low and Middle-Income Countries (LMICs) [9,17]. The questionnaires were translated into Luganda (the commonest local language in the locality of the study and Uganda). Research assistants administered the questionnaire to study participants face to face, in private rooms at the clinic, in either English or Luganda.

For the qualitative methods, we used FGD guides for adolescent girls living with HIV, in-depth interview guides for parents and key informant guides for health care providers. The Principal Investigator trained two social scientists as research assistants on conducting the qualitative discussions. The FGDs and in-depth interviews were conducted in Luganda or in English languages depending on what was convenient for the participants. Key informant interviews were conducted in English as all health workers could understand and speak English. All qualitative interviews and discussions were audio recorded and transcribed verbatim. The transcripts in Luganda were translated into English before analysis.

### Variables and measurements

The dependent variable was “uptake of HPV vaccine”, which was defined as the proportion of the participant who had received all the recommended three doses of the HPV vaccine. For women aged 15 years and older, and those immune-compromised and/or HIV-infected, receiving Highly Active Antiretroviral Therapy (HAART) or not, a 3-dose schedule (0, 1–2, 6 months) is recommended [18]. During data collection, information on uptake of HPV vaccination was obtained as follows: not vaccinated, received one dose, received two doses (second dose) and received three doses (third dose) of HPV vaccine. Participants who had received all the three doses were categorized as “fully vaccinated” and coded “1”, while the others who had received one or two doses (partially vaccinated) and those who had not received any dose were categorized as “not vaccinated” and coded “0” for analysis. The outcome of interest were adolescents who had received all the recommended three doses.

We determined socio-demographic characteristics of the participants and their caretakers including age, sex, education level and occupation status. Our independent variables included knowledge about the HPV vaccine, and health service-related factors. Knowledge about the HPV vaccine was measured as a composite variable by asking participants six questions about the HPV vaccine. This approach had been used in other studies to measure knowledge levels for HPV among adolescents, including studies in our settings [19,20]. Other health service-related factors included HPV vaccine information in the health education sessions, provider attitude and counseling skills and confidentiality observed, availability of Information, Education and Communication (IEC) Materials, and privacy at the health facility.

### Data analysis

Our quantitative data analysis comprised generating descriptive statistics and estimation of logistic regression models. We conducted descriptive analysis to generate frequencies and percentages of the outcome, socio-demographic and knowledge variables. Descriptive results were presented using frequencies and percentages or graphs. We then conducted bivariate and multivariable logistic regression analysis to determine crude and adjusted Odds ratios (OR), with 95% confidence intervals, of factors associated with completion of HPV vaccination among adolescent girls living with HIV. Adjusted odds ratios with p-value < 0.05 were considered statistically significant.

For qualitative data, audio records were transcribed and read through repeatedly for quality control, including ascertaining whether the patterns of the responses were complete and the responses captured in their complete form. Qualitative data was analyzed using a thematic approach with the help of NVivo.12 software. Transcripts were read by the Principal Investigator in order to familiarize with the data. The transcripts were coded to obtain emerging codes. These codes were refined through discussion of the emerging codes between the Principal Investigator, an analyst and study supervisors.

A second round of coding was done and a code structure generated. This code structure was applied to all codes by the Principal Investigator using NVivo.12 software. Subthemes and themes were then generated through cross-linking the data, code structure, emerging and unique issues and the stated events. The findings were integrated with quantitative data and direct quotes from the qualitative data were added to the results [21].

## Results

### Characteristics of study participants

A total of 264 adolescent girls aged 9–19 years were evaluated. We excluded three adolescent girls who declined to participate. The mean age of the participants was 14.4 (SD±2.8). Nearly half, 45.5% (120/264) of the adolescents were aged 13–16 years (Table 1). More than half of the participants, 58.7% (155/264) had attained a primary level of education, and 72.7% (192/264) resided in an urban area. About two-thirds, 65.1% (172/264) stayed with parents.

**Table 1:** Socio-demographic characteristics of the adolescent girls living with HIV aged 9–19 years at Mulago ISS clinic, Kampala.

*Variable*	*Frequency(n = 264)*	*Percentage (%)*
**Age groups (in years)**		
9–12	75	28.4
13–16	120	45.5
17–19	69	26.1
**Highest education level complete**		
No formal education	8	3.
Primary	155	58.7
Secondary	86	32.6
Tertiary	15	5.7
**Current education status**		
Enrolled at school	214	83.6
Not in school	50	16.4
**Residence**		
Urban	192	72.7
Rural	72	27.3
**Living arrangements**		
Stay with parents	172	65.1
Doesn’t stay with parents	92	34.9
**Relationship with the care taker, if not parents (n = 92)**		
Sibling	21	22.8
Grandparents	32	34.8
Uncle/aunt	36	39.1
Other*	3	3.3

### Prevalence of HPV vaccination

**Fig 1:**
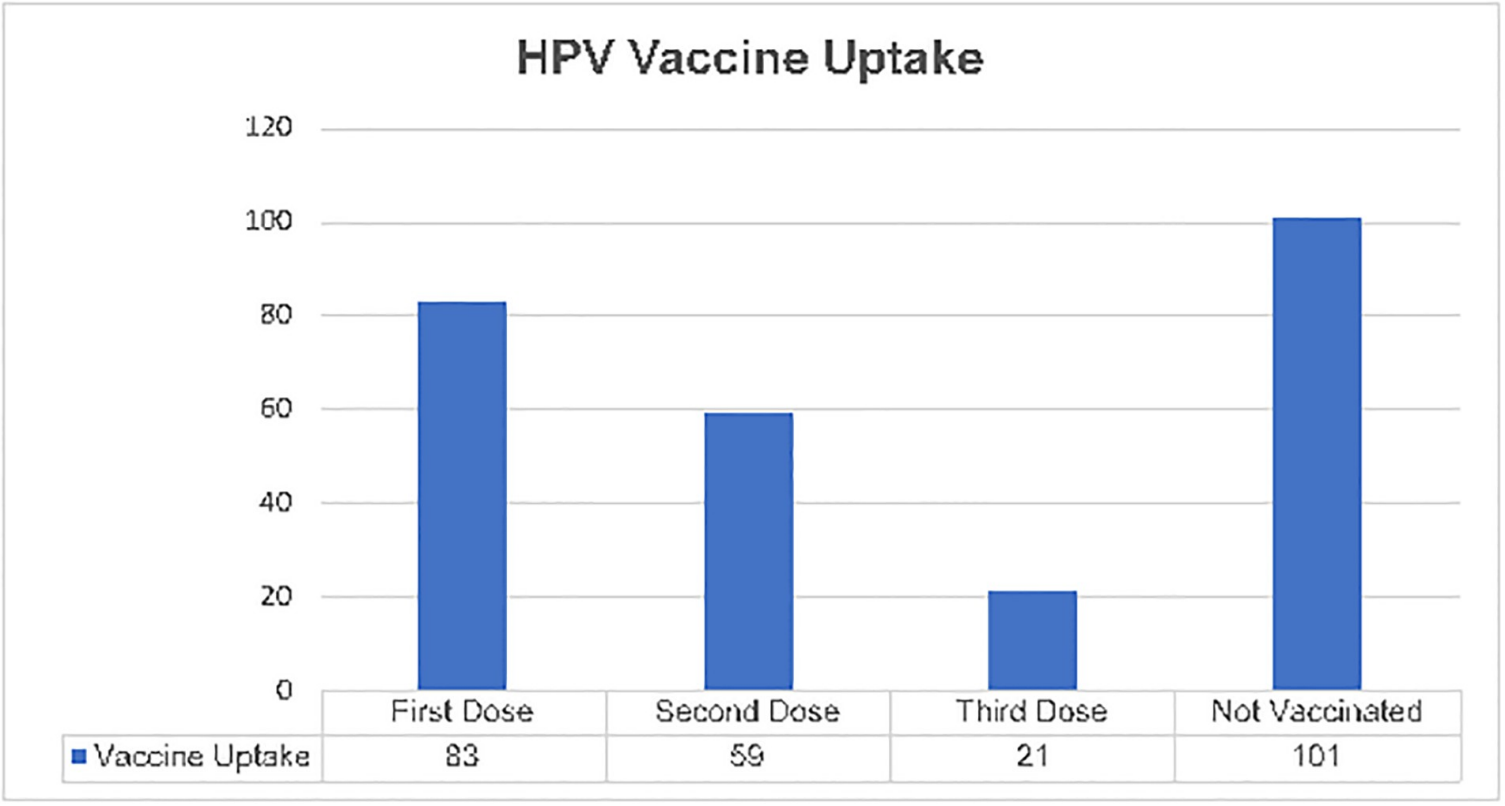
Prevalence of HPV vaccination among adolescents living with HIV aged 9–19 years.

Overall, only 8.0% (21/264) of the participants were fully vaccinated (received all the 3 doses) and 22% (59/264) had received at least two doses of HPV vaccine. Only a third, 31.4% (83/264) of the participants had received at least one dose of the HPV vaccine (Fig 1).

### Knowledge about HPV vaccination among adolescent girls living with HIV attending ISS clinic, Mulago

More than half, 59.1% (156/264), of the participants had ever heard of the HPV vaccine. Forty-six percent (121/264) of the adolescents did not know the benefits of HPV vaccination, and 74.6% (197/264) did not know the number of doses of the HPV vaccine. Close to a third, 29.2% (77/264) of the adolescents in this study did not know where to access the HPV vaccine (Table 2).

**Table 2:** Knowledge about HPV vaccination among adolescent girls living with HIV attending ISS clinic, Mulago.

*Topic*	*Frequency(n = 264)*	*Percentage (%)*
**Aware about HPV vaccine**		
No	108	40.9
Yes	156	59.1
**Benefits of HPV vaccination**		
Do not know the benefits	131	49.6
Prevent cervical cancer	133	50.4
**Number of doses of HPV vaccine**		
Do not know	197	74.6
Three	67	25.4
**Intervals between doses of the HPV vaccine**		
Do not know	260	98.5
Know (0, 1–2, 6 months)	4	1.5
**Target age group for the vaccine**		
Do not know	193	73.1
Between 9 and 19 years	71	26.9
**Qualification to get the HPV vaccine**		
Only male adolescents	4	1.5
Only female adolescents	156	59.1
Both male and female adolescents	47	17.8
Adults only	57	21.6
**Places that offer HPV vaccine** *		
Do not know	77	29.2
Mulago National Referral Hospital	125	47.4
Uganda Cancer Institute	20	7.6
Another health facility	42	25.9

*Multiple response questions.

### Sources of information about HPV vaccination

**Fig 2:**
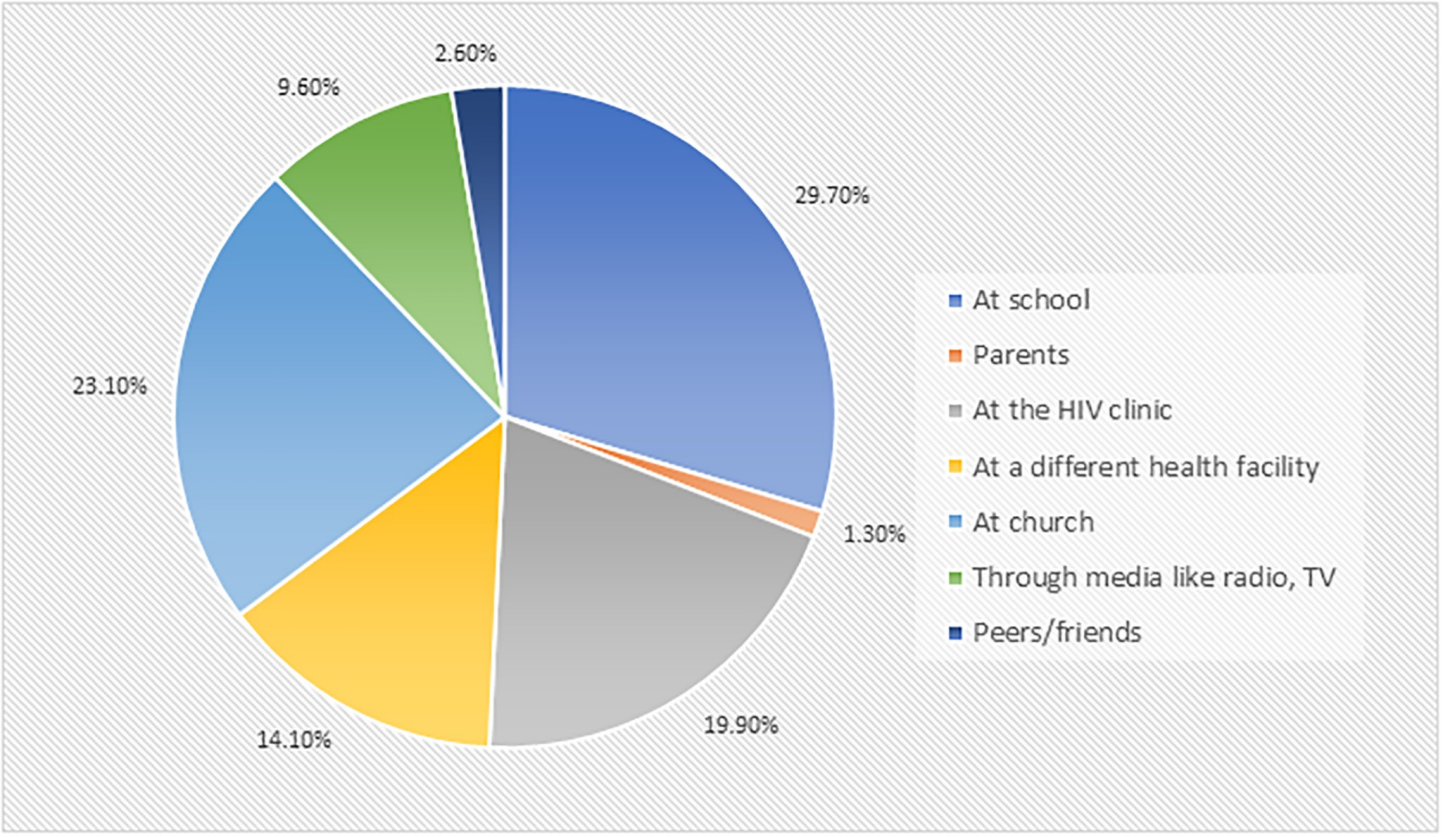
Source of information about HPV vaccination.

About a third of the participants, 29.7% (46/156) had heard about the vaccine from school (Fig 2). Minority of the participants 1.3% (2/156) had heard about HPV vaccination from their parents.

### Venues of first, second and third dose of HPV vaccination

**Fig 3:**
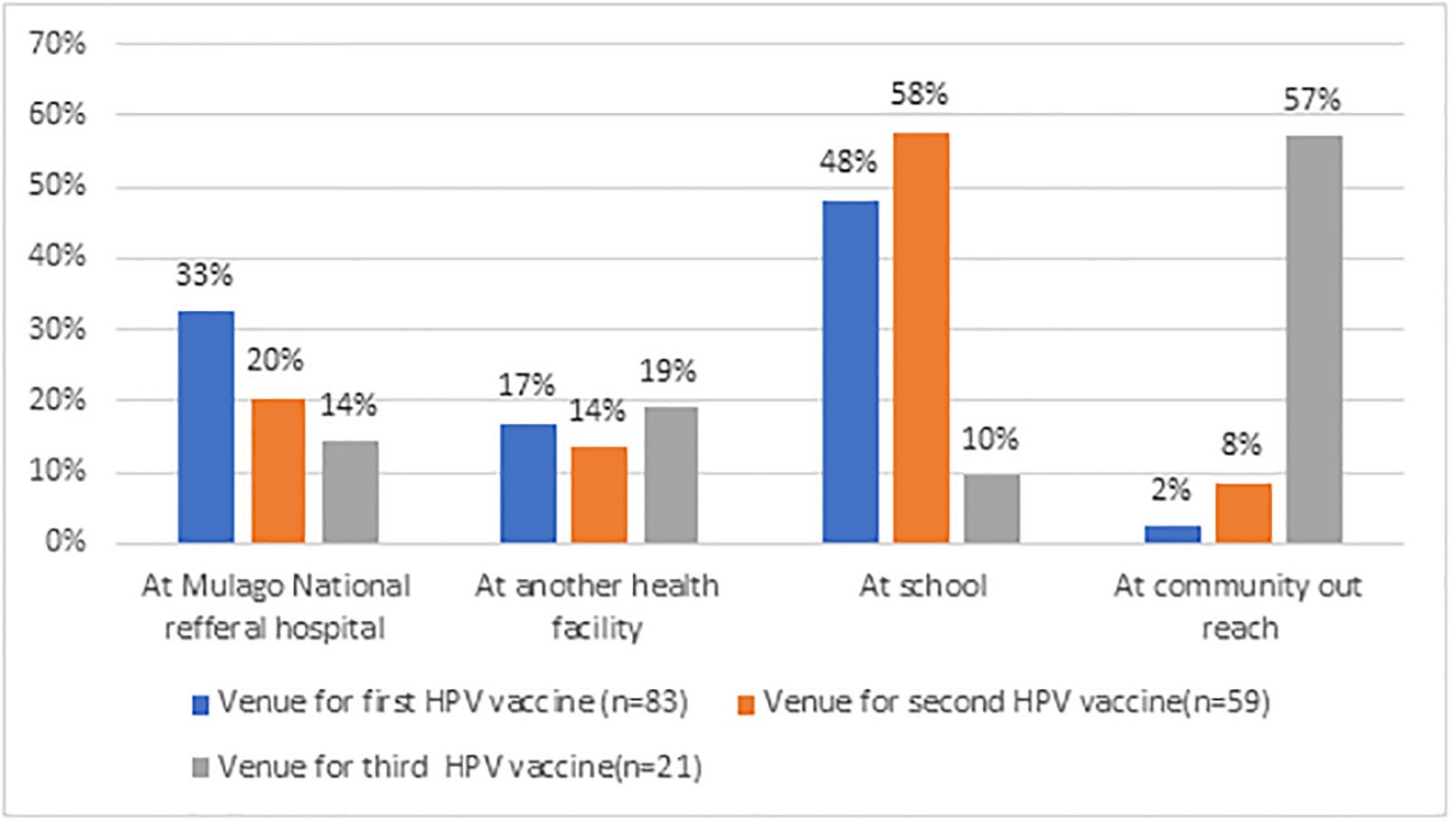
Venues of first, second and third HPV doses.

Overall, 48.19% (40/83) of the participants received their first dose of HPV vaccine from school while 57.6% (34/59) of the participants got their second dose of HPV vaccine from school. 57.1% (12/21) of the participants got their third dose from community out reaches (Fig 3).

### Factors associated with uptake of HPV vaccine among adolescent girls living with HIV aged 9–19 years

In the multivariable analysis, we adjusted for age of adolescent girls, age of caretakers, level of education, receipt of encouragement from health workers and receipt of encouragement from community members, and receipt of counseling from community members. Only the covariate “receipt of counseling from community members” was significantly associated with a three-fold likelihood to get fully vaccinated (COR = 3.28, CI = 1.07–10.08, P-value = 0.038) (Table 3).

**Table 3:** Bivariate and multivariable factors associated with uptake of HPV vaccine among adolescent girls living with HIV aged 9–19 years.

Variable	Fully vaccinated (n = 21)	Not fully vaccinated (n = 243)	COR (95% CI)	AOR (95% CI)
	Freq (%)	Freq (%)		
Age categories				
9–12	4 (19.1)	71 (29.2)	1	1
13–16	10 (47.6)	110 (45.3)	1.61 (0.49–5.34)	0.67 (0.14–3.17)
17–19	7 (33.3)	62 (25.5)	2.00 (0.56–7.17)	0.47 (0.07–3.31)
Education level				
No formal education	2 (9.5)	6 (2.5)	1	1
Primary	8 (38.1)	147 (60.5)	0.16 (0.03–0.94) *	0.22 (0.02–2.34)
Secondary	9 (42.9)	77 (31.7)	0.35 (0.06–2.00)	0.34 (0.04–3.21)
Tertiary	2 (9.5)	13 (5.4)	0.46 (0.05–4.10)	0.94 (0.05–17.75)
Age of the caretaker				
18–35	4 (19.1)	72 (29.6)	1	1
36–49	10 (47.6)	123 (50.6)	1.46 (0.44–4.84)	1.04 (0.27–3.97)
50+	7 (33.3)	48 (19.8)	2.63 (0.73–9.46)	2.52 (0.58–10.82)
Receipt of encouragement from a health worker at ISS clinic				
No	7 (33.33)	185 (76.13)	1	1
Yes	14 (66.67)	58 (23.87)	6.38 (2.46–16.56) *	2.69 (0.82–8.78)
Receipt of encouragement from a health worker elsewhere				
No	5 (23.81)	146 (60.08)	1	1
Yes	16 (39.92)	97 (39.92)	4.82 (1.71–13.58) *	2.7 (0.27–9.87)
Receipt of encouragement from community members				
No	6 (28.57)	176 (72.43)	1	1
Yes	15 (71.43)	67 (27.57)	6.57 (2.45–17.63) *	3.28 (1.07–10.08) *

* = Statistically significant (P-value less than 0.05); COR = Crude odds ratio; AOR = Adjusted odds ratio.

### Qualitative results

In the follow-up qualitative work, to understand explanations for the low uptake, we identified three major themes: (1): Limited information about HPV vaccination, (2) Parental influence on adolescent decisions and (3) Inadequacy of HPV vaccination services.

### Limited information about HPV vaccination

Adolescent girls reported that they did not have sufficient information about HPV vaccination particularly information about the number of doses for an adolescent living with HIV and the intervals when they are meant to receive the different doses. Although adolescents reported that there was healthcare education at the facility conducted by the adolescent peer supporter and other healthcare workers, in many instances, adolescents stated that they had not heard about HPV vaccine and did not know when and where to get the HPV vaccine. For example, in one FGD, adolescents agreed that:

***Participant 4****: “Me, I have never heard about HPV vaccination and that is why am not vaccinated*.

***Participant 6***: For me, *I don’t know if there is any hospital that gives HPV* [vaccine]. *No one has ever told me to get it*”

(FGD_1; 15–19 years).

Moreover, even the peer educators confessed that they lacked sufficient information about HPV vaccination and were therefore not in a good position to refer adolescents for this vaccination. When asked why adolescents are not being referred for the vaccine, an adolescent peer supporter reported:

*I think it’s because we lacked more information about it* [HPV vaccination]. *If we* [adolescent peer supporter] *had information about it, we would have referred many of the young girls to get vaccinated. The little information we have is what we are using to help the young girls. But if we get more information about HPV, we will refer many girls for the vaccine*”

(KI 2, Peer support).

In addition, parents to these adolescents also reported that they did not have the correct information about HPV vaccination. While some parents thought that this vaccine was not good for a person living with HIV, others were suspicious about the side effects of the vaccine. Consequently, some parents could not talk to their adolescent girls about HPV vaccination and in some cases, parents stated discouraging their adolescents from getting the vaccine. A mother indicated she did not know about HPV vaccination but knew about testing for cervical cancer:

*I haven’t heard much about vaccination. I usually hear about testing for cancer of the cervix. I didn’t even know that it* [cancer of the cervix] *can be vaccinated. So, I cannot even recommend a vaccination I don’t know about. What are its side effects? All I knew is the cancer of the cervix testing*.”

(IDI 3; Female).

There was therefore an information gap among the adolescents, their parents and some health workers concerning HPV vaccination.

Due to the inadequacy of the appropriate information about HPV vaccine, adolescents and the parents stated negative and false information included myths and misconceptions about the HPV vaccination. These included perceptions such as: “the vaccine has poisonous elements”, “vaccines cause diseases especially cancer”, “vaccination will lead to infertility” and that “vaccines are a trick to reduce the population growth rate”. An adolescent girl explained how the HPV vaccination would affect her normal body:

“*Yes, there are side effects, and it* [the vaccine] *might cause other illnesses or it* [the vaccine] *may result into one getting sick to an extent of her hand getting amputated*”

(P4: FGD 4; 15–19 years).

A mother to one of the unvaccinated girls stated some of the prevailing myths within her community:

*“…also, what we hear in the community is that if you vaccinate your child, she will become infertile and won’t be able to give birth in the future. This is because they* [community members] *believe that abazungu* [white men] *want to reduce the African population*”

(IDI 7; Female),

Consequently, this miss-information and myths culminated in fears and rejection of the vaccination as one adolescent girl explained:

“*People fear vaccines because they know once you are vaccinated, you acquire the virus*”

(FGD 1; 15–19 years).

### Parental influence on adolescent decisions

Adolescents reported that whenever they would get an opportunity to be vaccinated, they consulted and relied upon their parents to decide whether to get vaccinated or not. However, some responses from parents would cause adolescents to reject vaccination. For instance, parents who suspected negative outcomes of the HPV vaccine would not allow their children to be vaccinated. This was clearly brought out by a health worker who offers health talks to the adolescents:

“*I have heard two girls talking about their parents not wanting them to get the HPV vaccine. And when I ask them why their parents don’t want them to get the vaccine, they said their parents just say they don’t want them to get it. The parents say that the drugs might be expired*”

(KII 1; Health worker).

### Inadequacy of HPV vaccination services

Health workers at the ISS clinic reported that HPV vaccination was not a prioritized service at health facilities in the country. They stated that service provision at HIV clinic/ care points at health facilities mainly emphasized service provision for HIV and its opportunistic infections. Consequently, the adolescents were not told about the vaccine since HPV vaccination was not part of the routine services at the facility. One health worker noted how it was generally difficult for adolescents to access HPV vaccination services at HIV clinics:

… *the problem is access to this vaccine. We at the HIV clinics mainly treat HIV and maybe other infections like tuberculosis. There is no consistent supply of HPV vaccines to this clinic. So, how will the girls get this vaccine constantly? This also means the girls will not be able to complete the vaccination jabs simply because it is not a routine service*.

(KII 3; Adolescent Focal Person).

Finally, health workers and adolescents talked about school outreaches organized by the Ministry of health, which entailed HPV vaccination of adolescent girls. While the school outreaches would provide an alternative platform for HPV vaccination, these outreaches were reported to occur once or twice a year and students would not be equipped with prior information. Adolescents also reported that there would be no counseling provided to them prior to the vaccination. Hence adolescents reported being vaccinated without knowing what they were receiving and were also unaware of the number of doses they were supposed to receive. One adolescent reported how she was forcefully vaccinated:

“***P5***: *They* [Teachers] *commanded all the girls to get out of the class and we moved out*. So, *we got the injection just like that. When we asked the health worker what they were vaccinating us for, they told us it is a vaccine against a cancer. So, we* just got the vaccine.”

(FGD 2; 9-15YEARS).

## Discussion

In this study, we sought to determine uptake and factors associated with HPV vaccination among adolescent girls (aged 9–19 years) living with HIV in a low resource setting. We found a significantly low Uptake of HPV vaccination among this population, with most participants getting their third dose through community outreaches. Although knowledge about HPV vaccination was low among the adolescent girls living with HIV, schools were the major source of information and platform for HPV vaccination. Indeed, our follow-up qualitative findings showed that adolescent girls and their parents, expressed having inadequate information about HPV vaccination, yet adolescents’ decision to get the vaccine relied on their parents’ guidance.

Overall, less than a quarter of the participants had received all the recommended three doses of the HPV vaccine. The low uptake in this study corresponds with other studies conducted among the general population of adolescent girls (10 to 14 years) in Uganda, ranged between 16% and 43.3% across the geographical regions in Uganda [11,12,22]. This is in line with the UNICEF update of 2022 HPV vaccine protection among adolescents girls, which was at 14% [23]. All these prevalence still fall below the recommended national target of 80% HPV vaccinating for all eligible girls [24]. This was due to lack of right information about HPV vaccine which contributed to the poor health seeking behavior of the girls and their caretakers regarding the HPV vaccine as agreed upon by the qualitative data. The adolescents living with HIV are more at risk of cancer of the cervix hence are in more need of the HPV vaccine than the other adolescents’ girls but the level of HPV vaccine uptake in our study is at the lowest among the recorded researches in Uganda. The low uptake of HPV vaccine among the adolescent girls living with HIV will affect the government’s efforts of primary prevention of cancer of the cervix among patients living with HIV.

In both the quantitative and qualitative findings, schools were the most used venue for the first dose of HPV vaccine with nearly half of the study participants getting their first dose at school. This could be attributed to the ministry of health plan of conducting the HPV vaccine campaigns in schools [25]. However, majority of the participants got their third dose from community out reaches. This finding reveals a gap in the HPV vaccination implementation in schools, which focuses on two doses for all girls and omits the HIV positive group that requires a third dose (recommended for those living with HIV). Hence, adolescents living with HIV must outsource for the third dose and this is after being equipped with the knowledge on the required number of doses according to their sero-status. In addition to utilizing schools as a platform for HPV knowledge provision, they should be equipped with the capacity to provide the third dose, as a means of improving uptake among those who should get it. However, schools should avoid stigma and unnecessary disclosure of the HIV status of the adolescent girls while providing HPV vaccination.

In this study, most participants had received information about HPV vaccination from school. This could be a result of the Uganda Ministry of Health guideline of providing HPV vaccine information and immunization to adolescent and young women in schools and at health facilities that provide routine immunization services [25]. There is, however, no documented data about source of HPV vaccination information in Uganda and our study is the first to provide this information. About a half of the adolescents did not know the benefits of HPV vaccination. This was due to not discussing HPV vaccine as part of the health education talks. Our results agree with a study done by Rujumba et al, in the rural areas of Uganda which showed that majority of adolescents had inadequate knowledge about HPV vaccine [17].

We found that majority of the participants did not know the target age group for the vaccine as well as the required number of doses of the HPV vaccine. Similar studies, on HPV vaccination in Uganda, show that participants were not sure about the name of the vaccine they had received or the disease against which the vaccine protected, and the number of doses they were supposed to receive [11,12]. Close to a third, of the adolescents in this study did not know where to access the HPV vaccine. The participants in qualitative arm of the research agreed further by stating that they did not have sufficient information about the HPV vaccination particularly the information about the number of doses for an adolescent living with HIV and the intervals when they are meant to receive the different doses. This is in line with a study conducted by, Nabirye et al among adolescents’ girls aged 9–15 in Mbale which reported low levels of awareness about HPV vaccine among adolescents and young women in peri-urban Uganda [9].

About a quarter of the participants had been health educated by a health worker at the Mulago ISS clinic to go for HPV vaccination. This was due to health workers not being equipped with the required information to pass on to the adolescents as per responses from the qualitative interviews. Our findings concur with those of a study conducted in Oyam District, Northern Uganda which showed healthcare workers and VHTs who are the frontline workers for the vaccination program had gaps in knowledge about the vaccine, side effects and the national HPV vaccination policy hence didn’t pass on the information to the adolescents [17].

Parents had a great influence on the decision an adolescent made concerning HPV vaccination. This is in line with a study conducted in province of Québec Canada by Andrea Krawcyzk et al which showed 88.2% of the parents accepted the HPV vaccine on behalf of their children and 11.8% declined the HPV vaccine on behalf of their children [26].

Our findings showed that receipt of encouragement from community members (village health teams and church leaders) was significantly associated with a three-fold likelihood to get fully vaccinated compared to those who did not get any encouragement from the community. A study conducted in a rural setting among adolescents aged 12–17 years was in line with our findings; The prevalence of uptake of HPV vaccine was three times higher among adolescents who had been encouraged by a village health team member (VHT) to go for HPV vaccination compared to those who had not been encouraged by a VHT [25].

Our study should be considered with some limitation. Our study was in an HIV clinic that provides HIV services including cervical cancer screening services and health education on HPV vaccination. Therefore, the findings may not be generalized to adolescents living with HIV who are not enrolled in HIV care. However, we followed the WHO guideline for HPV vaccination among immune suppressed adolescents to define and measure the outcome (full HPV vaccination). Thus, the prevalence of the outcome is based on an internationally recognized guideline and measurement, which may allow for comparability of results across settings where the WHO guidelines apply (because we are using the same international guideline).

## Conclusions

There was a low uptake of HPV vaccination among adolescents and young women living with HIV, which warrants a need to integrate information of HPV vaccination into the routine health education sessions in schools and health facilities offering routine HIV care. Integration of HPV vaccination services into routine HIV care should be considered. This will make it a one stop center. Community members should be part of the team recognized in disseminating information about HPV vaccination since they play a significant role of counseling and mobilizing adolescents to receive the HPV vaccine.

There was a general lack of the right information about HPV vaccination among adolescents and parents, which gave room to unawareness and misconceptions about the vaccine. Despite having limited knowledge about HPV vaccination, parents had a great influence on the decision an adolescent made concerning HPV vaccination. Both health facility and school-based HPV vaccination services were inadequate for this population.

To improve awareness and uptake of HPV vaccination to the recommended ministry of health target of 80%, there is a need to strengthen the health education campaigns, and resourcing of the health facilities to provide vaccination services both at the health facilities and through outreaches.

## Supporting information

S1 Checklist (DOCX)

S1 Dataset (CSV)
