# Supplementary material for: Uptake of human papilloma virus vaccination among adolescent girls living with HIV in Uganda: A mixed methods study
Source: PLoS One. 2024 Aug 8;19(8):e0300155. doi: 10.1371/journal.pone.0300155 (PMC11309443; doi:10.1371/journal.pone.0300155)
Supplement: Attachment — Submitted filename: Response letter _ draft 2-MM.docx [file pone.0300155.s003.docx]

August 27^th^, 2023.

Dear Editor,

Thank you for considering our manuscript, “*Uptake of Human Papilloma Virus Vaccination Among Adolescent Girls Living with HIV in Uganda: A Cross Sectional Study*” for publication in *PLOS ONE*. Below, we describe how we have further strengthened the manuscript in response to the journal requirements and reviewers’ comments. We quote reviewer comments in *italics*, and changes to the original manuscript are indicated in text. Pages and line numbers referred to herein are in the “revised manuscript with tracked changes”.

**ACADEMIC EDITOR:**

***Comment:*** *The manuscript reports about an important area in HPV vaccination especially from the context of low-resource settings. During revision, the authors should clearly explain how those participants that received one or two HPV doses were categorized. Was the outcome of interest three doses?*

**Response:** Thank you for this concern for clarity on the categorization of the participants regarding the outcome. Categories were as follows: 1. Participants who received ALL the three doses of HPV vaccination were categorized as “fully vaccinated”; 2. Participants who had received one or two doses were categorized as “partially vaccinated” and 3. Participants who had not received any vaccination were categorized as “not vaccinated”. Since our outcome of interest was receipt of the three doses, the participants were then categorized into two as “*Fully vaccinated*” coded “1” and other participants as “*partial or not vaccinated”* (coded “0”). This explanation has been added to the main manuscript body on page 10 lines 185-189.

Also, the term “*partial or not vaccinated*” is used in the manuscript to refer to those who received partial or no vaccination.

**Reviewer #1:**

***Abstract***
***Comment:*** *Background. It would be good to give the gap/problem statement/rationale of the study among HIV infected adolescent and proceed with the objective. An appreciation of the gap is key while reading the abstract is key.*

**Response:** *We have added a gap/problem statement to the background of the abstract before stating the objective* (Page 3; line 27 to 30).

***Comment:*** *Methods. Does it mean uptake which I think is your main outcome was defined as “A participant who had received all the three recommended HPV vaccine doses” living out those who received one and two does. If yes, justify why you defined your outcome like that? How did you categorized those who received one, or two dozes? They can’t be categorized as not-vaccinated. Please note, not fully (partial) vaccinated is different as not vaccinated”*
**Response**: According to the World Health Organization (WHO) guidelines on HPV vaccination, adolescents aged 15 years and above, and those immune-compromised and/or HIV-infected, (whether receiving Highly Active Antiretroviral Therapy (HAART) or not) should receive a 3-dose schedule (0, 1–2, 6 months) of HPV vaccine to be fully vaccinated. This informed the outcome of our study. This explanation is in the methods section page 10 lines 180-182

We have also indicated that the participants were in three categories: fully vaccinated; partially vaccinated; and not vaccinated. (page 10 line 185-188). However, since outcome of interest was “fully vaccinated” we coded it as “1” and considered the other two categories as “*partial or not vaccinated” (coded as “0”) in order to run the analysis and obtain factors that were associated with completion of HPV vaccination among adolescent girls living with HIV.*

***Comment: Results****: Results does not clearly show the factors associated with full vaccination in the logistic model. Can you list all the co-variates that were associated with full vaccination, add COR, P-Value and 95% CI*
**Response:** Our logistic regression model included the following variables: age, education level, age of caretaker, Receipt of encouragement from a health worker at the HIV clinic, Receipt of encouragement from a health worker elsewhere and Receipt of counseling from community members. Only one variable/ covariate was significant in this model: received counseling from community members (adjusted OR 3.28, Cl:1.07-10.08, P=0.038). This result is reported in the abstract with its corresponding confidence interval (page 3 to 4; line 47 to 49).

***Conclusion:*** *Your objective was uptake and factors associated with vaccination. Can you define uptake as any person who received HPV vaccine which will be more than 10% and will require a poison regression model as opposed to logistic regression? Your current conclusion of HPV vaccination, not fully vaccination or you wanted to say that “Full HPV vaccination was low among adolescent girls living with HIV”?*
**Response:** We have edited the statement to state, “Full HPV vaccination was low among adolescent girls living with HIV” because our outcome of interest was full HPV vaccination. Therefore, since the prevalence of “Full vaccination” was at 8%, which is less than 10%, we will not need to run a modified poison regression model. Page 13 line 226-228

***Comment:*** *Main manuscript line 97-100 “**Understanding the prevalence of HPV vaccination and associated factors among adolescents’ girls living with HIV will inform the development of targeted strategies to scale up this intervention to this patient population that is at a higher risk of cancer of the cervix. How will this cross-sectional study lead the development of strategies of scale HPV?*

**Response:** We would like to clarify the statement by rephrasing it to state that “Understanding the prevalence of HPV vaccination and associated factors among adolescent girls living with HIV will identify the unmet need for HPV vaccination among adolescents living with HIV and lay the foundation for developing strategies to scale up this intervention to this patent population that is at a higher risk for cancer of the cervix”. This does not mean that it will lead to the development of strategies. However, the study results provide information that can be used to identify the gap and create the basis for scale up uptake of the HPV vaccination. We did not consider the word “*lead*” in this statement as it would mean something else. Page 6 line 107-109

***Comment: Results:*** *line 241 to 250 “In the multivariable analysis, after adjusting for age of adolescent girls, age of caretakers, level of education, receipt of encouragement from health workers and receipt of encouragement from community members, receipt of encouragement from community members was significantly associated with a three-fold likelihood, (COR=3.28, CI=1.07-10.08, P-value=0.038), to get fully vaccinated compared to those who did not get any encouragement from the community (Table 3). Do you mean all the co-variates; age of adolescent girls, age of caretakers, level of education, receipt of encouragement from health workers and receipt of encouragement from community members, receipt of encouragement had the same COR, P value and 95% CI? Or each of covariates had COR, P-Value and 95% CI. If yes, include those in each co-variate.*
**Response:** Thank you for bringing this out. We had not clearly stated the result from the multivariable analysis. We have rephrased this result to show that only one covariate was significant in the model as stated: “In the multivariable analysis, we adjusted for age of adolescent girls, age of caretakers, level of education, receipt of encouragement from health workers and receipt of encouragement from community members, and receipt of counseling from community members. Only the covariate “receipt of counseling from community members” was significantly associated with a three-fold likelihood to get fully vaccinated.” (page 15 lines 255-260)

***Comment:*** *Any strength to your study? If there, add. But the main issue is outcome definition fully vaccination vs partial vaccination vs no vaccination.*

**Response:** we have added a strength to the manuscript body (Page 19-20; lines330-335). Indeed, the definition of our study was based on the WHO guidelines for HPV vaccination among immune suppressed adolescents. This means the outcome result can be compared with other outcome results that use the WHO guideline to define and measure their HPV vaccine uptake among immune suppressed adolescents.

**Reviewer#2: INTRODUCTION/BACKGROUND**
***comment****: The introduction and background have failed to mention or has omitted latest data on HPV vaccine from the current statistics at MOH Uganda and recent publications in this topic. This section needs rewriting and updating recent available data to uptake and utilization of HPV vaccine in Uganda.*

**Response:** Thank you for noticing this gap. We have updated the latest research done by Lydia et al at Mulago national referral hospital two years after the national rollout of HPV vaccination Programme in Uganda. The study found a completion level of the second dose of HPV vaccine within 6 months of the first dose at 43.3%. The WHO recommends a 2-dose schedule for immunization of immune-competent girls aged 9–14 years. This information has been added to strengthen the background. Page 6 line 96-99

**Note**; a research done by Lydia Patrick et al was done in an adolescent clinic in a national referral hospital yet ours was done in a specialized HIV clinic

***Comment****: In 2022, WHO and UNICEF released updated data on performance of immunization post pandemic, with newer information, especially reflecting on how HPV vaccine, one of the 4 main affected vaccines are currently performing. This paper has completely ignored that data, and as such builds its case on outdated and NON-mentioned data thus doing a lot of disservice to a very important topic. The result is a poorly developed case for such an important topic and subject.
This section needs to be redone.*
**Response**: We have updated the required information into the manuscript. As per the UNICEF website, by 2022, the HPV vaccine had been fully introduced in 130 countries. However, only 14% of girls are currently fully protected against HPV, indicating the need for increased efforts in this area. Page 6-line 101-103

**METHODS**
***Comment:*** *The description of the conduct of the study is very brief and very shallow. What was done from the time an adolescent walked into the clinic, was offered a chance to participate, was selected… and participated to the end … this procedure has not been described. Brief mentions have been made, leaving the reviewer unable to effectively critic the sampling procedure and data collection exercise. The writers are encouraged to provide a more detailed description of how the study was conducted.*

**Response:** We have made additions to the way participants were practically selected, approached and invited to the interviews. A detailed description is included in the manuscript page 9 line 158-167

***Comment:*** *The biggest weakness of this study, that makes it fall short of a publication at the level of Plos one, is the decision of the authors to limit themselves to a quantitative study when they had a chance for a mixed methods study, through triangulation, and the unique opportunity to interview the adolescents, and invite them for focus group discussions and even conduct key informant interviews for focal health workers at the clinic and at the ministry of health. THIS constitutes a major omission and lost opportunity, that NOT ONLY weakens this study significantly, but also denies the authors an opportunity to add value to the study. This omission, has greatly weakened this study’s ability to make far reaching policy recommendations that can be adopted by the ministry of health and policy makers. This left them with a basic descriptive study, with limited experimental design use, with an attempt at logistic regression that was sop narrow as to reveal any new factors associated with low vaccine uptake and that failed to even affirm previously demonstrated factors from other similar studies in the same context. I think this failure of reproducibility of previous findings arose from a defect in the methodology and design of this study. Can this be cured or remedied and somehow make this study stronger? I do not have the answer to this.
The logistic regression, if it was done, was performed incorrectly. The factors did not yield any significant results, and even did not confirm or reject earlier study findings in this area. A posson regression would have better, but this is not possible because the factor spread and analysis frame designed by the authors was very narrow. The only way to cure this problem would have been use of triangulation, where the qualitative arm would have solved the major weaknesses and shortcomings of the quantitative arm. This was not done, thus leaving the study significantly weakened. Why the study opted to focus on uptake and omitted aspects and drivers of completion of HPV vaccination is not clear. Initiatives around addressing HPV vaccine failure must focus more on completion if uptake is to be of any value. This was another major omission of this study. One that at this stage may not be redeemed, yet largely undermines the utility of this study. Descriptive statistics alone at this level is not adequate.*

**Response:** Thank you for this comment. We think the reviewer is interested in two major issues: 1- adding a qualitative component to triangulate with the quantitative results. 2- why didn’t we focus on vaccine completion instead of uptake, which would affect the reproducibility of the recent research. (Hence some errors in the methodological approach). Our response to these issues is as follows:

- This was a baseline study in a unique population of adolescent girls living with HIV and having a different measure for the outcome indicator. Since it was a baseline in a special population, reproducibility would be low because the other studies used the second dose as the measure of completion, and we used the third dose as per the WHO guidelines. We therefore acknowledge that the difference in the results is due to the difference in the measurements (second dose Verses third dose). However, we estimated completion of the three doses to indicate uptake of HPV vaccine. (Our outcome is thus, completion of the three doses and not merely only uptake of HPV vaccination.
- Concerning the missed methods approach. We indeed did a follow up study which was a qualitative study to explain the results from the quantitative study. However, the qualitative study findings would overshadow the key findings in this quantitative study, which are crucial to note:

a) low uptake and completion of HPV vaccination among the adolescent girls living with HIV, (less than that among the general population of adolescent girls), This indicates that many adolescent girls living with HIV get partial doses and majority are not vaccinated, hence all do not mount an adequate immune response.
b) Low levels of knowledge about HPV vaccination among this population, implying a need to increase awareness and valuable information about the vaccination in this population. Schools, which were the largest source of information can be utilized to increase this knowledge.
c) Community health counsellors are a suitable vehicle to improve uptake and completion of HPV vaccination among this population in the Ugandan context.

Therefore, the difference in results include completion of Vaccination and the difference in outcome when compared to other studies, (which used a different measure), does not negate the rigor of the methods. The results from the other studies, which the reviewer referred to, are of a general population of adolescent girls (both HIV negative and HIV positive), hence they are of a mixed population and could potentially be confounded. Our study population was adolescent girls living with HIV.

*DISCUSSION, CONCLUSSION AND RECOMMENDATIONS
The discussion is limited at narrow. This is because of several reasons:
1. The narrow approach taken in background and introduction that omitted the broad concerns and recent data around HPV vaccination globally and Uganda in particular.*

**Response:** We have made additions to the discussion section.

2. The very narrow methodology avoiding a mixed methods approach that denied the authors the broad premise on which to make robust and cogent recommendations.

**Response:** As noted, the quantitative results have potentially sufficient key information that should not be overshadowed or under looked. We believe this baseline / formative study can be a basis for further inquiry into this vulnerable population of adolescents living with HIV in Uganda and similar settings.

*3. The failure of the authors to integrate the current post pandemic context and IA 2030 recommendations and targets into improving HPV vaccine uptake. These have completely been omitted or ignored by the authors. In the process, they have greatly weakened their own study.*

Response: Thank you for noting this. We would like to differ from this comment by noting that the study was done during the pandemic (even after the first wave of the pandemic in Uganda). Therefore, our results depict the current implementation of HPV vaccination including the guidelines and the post pandemic healthcare provision in Uganda. Moreover, the findings are in lieu of the Ugandan context, inherent with the customs of healthcare provision, peoples’ living and livelihood and the environmental factors that impact access, availability and utilization or uptake, including adherence to healthcare services particularly HPV vaccination. That’s why our results depict the importance of community counselling as opposed to the adaptability of technology (AI), which may not, currently and in the near future, be applicable in the Ugandan context.

Therefore, we did not omit or ignore the trends outside our settings. Rather than importing recommendations from highly resourced settings, we reported and recommend what is in the local setting of Uganda and similar low resources countries.

***Reviewer #3:*** *This paper looks at completeness of HPV immunization and HPV knowledge in HIV+ females ages 9-19.*

Comment: HPV vaccine is given in schools as 2 doses. HIV+ girls therefore did not receive a 3rd dose in schools in Uganda. So, the observations would in many ways be self-evident.

**Response:** Thank you for bringing this out. Schools are supposed to follow the World Health Organization guidance and the Uganda Ministry of Health, but the findings showed that they give two doses hence the adolescents living with HIV could not receive all the required doses of HPV vaccine at school. This finding reveals a gap in the HPV vaccination implementation in schools, which focuses on two doses for all girls and omits the HIV positive group that requires a third dose. This can potentially affect the ability of girls living with HIV to mount an adequate immune response. This shows that the schools are not abiding by the national guideline guidance.

***Comment:*** *These are girls receiving HIV care in an HIV clinic. Why not give the vaccine in the clinic? That would solve a lot of problems.*

**Response:** The package offered at the HIV clinic does not include HPV vaccination. HIV clinics mainly offer HIV treatment and prevention strategies plus treatment and prevention of opportunistic infections. HPV vaccination services are offered only in hospitals that offer vaccines against other diseases as per the child immunization card. In this manuscript, we have also recommended integration of HPV vaccination services into the routine HIV care. This will enable the adolescents living with HIV to receive the full course of the HPV vaccination timely. Page 7 line 123-125

***Comment:*** *Tables and Figures. Much of the same information is in the text so some of these are redundant.*

**Response:** We have edited some of the texts to remove redundant words/ results. We have made the narratives precise to communicate only the relevant results.

***comment:*** *Table 2. School not included.*

**Response**: Table 2 presents results about “knowledge on HPV vaccine” so schools where not a variable on the knowledge questions. However, “school” is included in the Figure 2, where we report about “sources of knowledge about HPV vaccine”.

***Comment:*** *Not sure what is meant by "cates" on line 275.*

**Response:** Thank you for this concern. I would like to apologize on behalf of the entire team for the typo it was meant to be caters not cates. A correction has been made on page 18 line 288

***Comment****: The authors need to put forward some solutions. A third dose in schools would stigmatize by inferring that the recipient has HIV.*

**Response:** Thank you for this guidance. Some of the young adolescents aged nine to ten years have not yet had full disclosure of the HIV status. They cannot tell the immunisation team that they are HIV positive to enable them get the adequate number of doses. We had made a recommended suggesting integration of HPV vaccination services into the routine HIV care for adolescents. This will help save the mistake of abrupt or unintended discloser of the HIV status of the adolescents to their peers and schools. Page 20 lines 340

***Comment:*** *How do results compare with HIV- girls?*

**Response**: To the best of our knowledge and literature searches, there is no documented study about HPV vaccination in only HIV negative girls that we found. The available studies do not have HIV status as a variable to distinguish a given population. These studies were conducted among the general population having both HIV negative and HIV positive adolescents. So, we cannot compare results for those who are HIV positive and those who are HIV negative.
