# Supplementary material for: Uptake of human papilloma virus vaccination among adolescent girls living with HIV in Uganda: A mixed methods study
Source: PLoS One. 2024 Aug 8;19(8):e0300155. doi: 10.1371/journal.pone.0300155 (PMC11309443; doi:10.1371/journal.pone.0300155)
Supplement: Attachment — Submitted filename: Response to Reviewers 22.01.docx [file pone.0300155.s004.docx]

20^th^ / 01/2024

Dear Editor,

Thank you for considering our manuscript, “Uptake of Human Papilloma Virus Vaccination Among Adolescent Girls Living with HIV In Uganda: A mixed method Study” for publication in PLOS ONE. Below, we describe how we have further strengthened the manuscript in response to the journal requirements and reviewers’ comments. We quote reviewer comments in italics, and changes to the original manuscript are indicated in text. Pages and line numbers referred to herein are in the “revised manuscript with tracked changes”.

***Editors comment;*** *The revised paper is indeed very strong. The qualitative findings included do provide more rigor to the study. Some minor observation that should be addressed by the authors: In the results section of the Qualitative findings, Line 335, the naming of the themes should be revised. Currently, the three themes are named with very long sentences that read like just descriptions. The name of a theme should be shorter and concise and the description of the theme can come just after the name.*

**Response**; thank you for the guidance. We have reduced on the length of the sentences for the themes and adopted;

(1): Limited information about HPV vaccination,

(2) Parental influence on adolescent decisions and

(3) Inadequacy of HPV vaccination services.

(Page 4 line 48-50, page 20 line 334-336)
