# Supplementary material for: Uptake of human papilloma virus vaccination among adolescent girls living with HIV in Uganda: A mixed methods study
Source: PLoS One. 2024 Aug 8;19(8):e0300155. doi: 10.1371/journal.pone.0300155 (PMC11309443; doi:10.1371/journal.pone.0300155)
Supplement: Peer review history

# Peer review: Decision Letter 0

**DOI:** 10.1371/journal.pone.0300155.r001

**Authors:** Aloysius Gonzaga Mubuuke

24 Jul 2023

PONE-D-23-17190UPTAKE OF HUMAN PAPILLOMA VIRUS VACCINATION AMONG ADOLESCENT GIRLS LIVING WITH HIV IN UGANDA: A CROSS SECTIONAL STUDYPLOS ONE

Dear Dr. Nakibuuka,

Thank you for submitting your manuscript to PLOS ONE. After careful consideration, we feel that it has merit but does not fully meet PLOS ONE’s publication criteria as it currently stands. Therefore, we invite you to submit a revised version of the manuscript that addresses the points raised during the review process.

==============================

ACADEMIC EDITOR: The manuscript reports about an important area in HPV vaccination especially from the context of low-resource settings. During revision, the authors should clearly explain how those participants that received one or two HPV doses were categorized. Was the outcome of interest three doses?==============================

Please submit your revised manuscript by Sep 07 2023 11:59PM. If you will need more time than this to complete your revisions, please reply to this message or contact the journal office at plosone@plos.org. When you're ready to submit your revision, log on to https://www.editorialmanager.com/pone/ and select the 'Submissions Needing Revision' folder to locate your manuscript file.

Please include the following items when submitting your revised manuscript:

- A rebuttal letter that responds to each point raised by the academic editor and reviewer(s). You should upload this letter as a separate file labeled 'Response to Reviewers'.
- A marked-up copy of your manuscript that highlights changes made to the original version. You should upload this as a separate file labeled 'Revised Manuscript with Track Changes'.
- An unmarked version of your revised paper without tracked changes. You should upload this as a separate file labeled 'Manuscript'.

If you would like to make changes to your financial disclosure, please include your updated statement in your cover letter. Guidelines for resubmitting your figure files are available below the reviewer comments at the end of this letter.

If applicable, we recommend that you deposit your laboratory protocols in protocols.io to enhance the reproducibility of your results. Protocols.io assigns your protocol its own identifier (DOI) so that it can be cited independently in the future. For instructions see: https://journals.plos.org/plosone/s/submission-guidelines#loc-laboratory-protocols. Additionally, PLOS ONE offers an option for publishing peer-reviewed Lab Protocol articles, which describe protocols hosted on protocols.io. Read more information on sharing protocols at https://plos.org/protocols?utm_medium=editorial-email&utm_source=authorletters&utm_campaign=protocols.

We look forward to receiving your revised manuscript.

Kind regards,

Aloysius Gonzaga Mubuuke

Academic Editor

PLOS ONE

Journal Requirements:

When submitting your revision, we need you to address these additional requirements.

1. Please ensure that your manuscript meets PLOS ONE's style requirements, including those for file naming. The PLOS ONE style templates can be found at 

https://journals.plos.org/plosone/s/file?id=wjVg/PLOSOne_formatting_sample_main_body.pdf and 

https://journals.plos.org/plosone/s/file?id=ba62/PLOSOne_formatting_sample_title_authors_affiliations.pdf

2. Please note that in order to use the direct billing option the corresponding author must be affiliated with the chosen institute. Please either amend your manuscript to change the affiliation or corresponding author, or email us at plosone@plos.org with a request to remove this option.

3. PLOS requires an ORCID iD for the corresponding author in Editorial Manager on papers submitted after December 6th, 2016. Please ensure that you have an ORCID iD and that it is validated in Editorial Manager. To do this, go to ‘Update my Information’ (in the upper left-hand corner of the main menu), and click on the Fetch/Validate link next to the ORCID field. This will take you to the ORCID site and allow you to create a new iD or authenticate a pre-existing iD in Editorial Manager. Please see the following video for instructions on linking an ORCID iD to your Editorial Manager account: https://www.youtube.com/watch?v=_xcclfuvtxQ

4. Please amend the manuscript submission data (via Edit Submission) to include authors Dr. Martin Muddu, Dr. Jean Pierre Kraehenbuhl, Dr. Caroline Birungi, Dr. Fred C. Semitala, and Dr. Andrew K. Tusubira.

5. Your ethics statement should only appear in the Methods section of your manuscript. If your ethics statement is written in any section besides the Methods, please move it to the Methods section and delete it from any other section. Please ensure that your ethics statement is included in your manuscript, as the ethics statement entered into the online submission form will not be published alongside your manuscript. 

Additional Editor Comments:

The paper should be proof-read by a more experienced writer to improve the language.

[Note: HTML markup is below. Please do not edit.]

Reviewers' comments:

Reviewer's Responses to Questions

**Comments to the Author**

1. Is the manuscript technically sound, and do the data support the conclusions?

The manuscript must describe a technically sound piece of scientific research with data that supports the conclusions. Experiments must have been conducted rigorously, with appropriate controls, replication, and sample sizes. The conclusions must be drawn appropriately based on the data presented. 

Reviewer #1: No

Reviewer #2: Partly

Reviewer #3: Yes

**********

2. Has the statistical analysis been performed appropriately and rigorously? 

Reviewer #1: No

Reviewer #2: I Don't Know

Reviewer #3: I Don't Know

**********

3. Have the authors made all data underlying the findings in their manuscript fully available?

The PLOS Data policy requires authors to make all data underlying the findings described in their manuscript fully available without restriction, with rare exception (please refer to the Data Availability Statement in the manuscript PDF file). The data should be provided as part of the manuscript or its supporting information, or deposited to a public repository. For example, in addition to summary statistics, the data points behind means, medians and variance measures should be available. If there are restrictions on publicly sharing data—e.g. participant privacy or use of data from a third party—those must be specified.

Reviewer #1: Yes

Reviewer #2: Yes

Reviewer #3: Yes

**********

4. Is the manuscript presented in an intelligible fashion and written in standard English?

PLOS ONE does not copyedit accepted manuscripts, so the language in submitted articles must be clear, correct, and unambiguous. Any typographical or grammatical errors should be corrected at revision, so please note any specific errors here.

Reviewer #1: Yes

Reviewer #2: Yes

Reviewer #3: Yes

**********

5. Review Comments to the Author

Please use the space provided to explain your answers to the questions above. You may also include additional comments for the author, including concerns about dual publication, research ethics, or publication ethics. (Please upload your review as an attachment if it exceeds 20,000 characters)

Reviewer #1: Thank you for the opportunity to review this article.

Violet Nakibuuka et al determined the uptake of, and factors associated with

Human Papilloma Virus (HPV) vaccination among adolescent girls living with Human Immunodeficiency Virus (HIV) attending a large urban HIV clinic in Uganda and concluded that HPV vaccination was low among adolescent girls living with HIV. However, I have few clarification comments

Abstract

Background. It would be good to give the gap/problem statement/rationale of the study among HIV infected adolescent and proceed with the objective. An appreciation of the gap is key while reading the abstract is key

Method. Does it mean uptake which I think is your main outcome was defined as “A participant who had received all the three recommended HPV vaccine doses” living out those who received one and two does. If yes, justify why you defined your outcome like that? How did you categorized those who received one, or two dozes? They can’t be categorized as not-vaccinated. Please note, not fully (partial) vaccinated is different as not vaccinated”

Results: Results does not clearly show the factors associated with full vaccination in the logistic model. can you list all the co-variates that were associated with full vaccination, add COR, P-Value and 95% CI

Conclusion: Your objective was uptake and factors associated with vaccination. Can you define uptake as any person who received HPV vaccine which will be more than 10% and will require a poison regression model as opposed to logistic regression? Your current conclusion of HPV vaccination, not fully vaccination or you wanted to say that “Full HPV vaccination was low among adolescent girls living with HIV”?

Main manuscript line 97-100 “Understanding the prevalence of HPV vaccination and

associated factors among adolescent girls living with HIV will inform the development of targeted strategies to scale up this intervention to this patent population that is at a higher risk for cancer of the cervix. How will this cross-sectional study lead the development of strategies to scale HPV vaccination?

Results: line 241 to 250 “In the multivariable analysis, after adjusting for age of adolescent girls, age of caretakers, level of education, receipt of encouragement from health workers and receipt of encouragement from community members, receipt of encouragement from community members was significantly associated with a three-fold likelihood, (COR=3.28, CI=1.07-10.08, P-value=0.038), to get fully vaccinated compared to those who did not get any encouragement from the community (Table 3). Do you mean all the co-variates; age of adolescent girls, age of caretakers, level of education, receipt of encouragement from health workers and receipt of encouragement from community members, receipt of encouragement had the same COR, P value and 95% CI? Or each of covariates had COR, P-Value and 95% CI. If yes, include those in each co-variate.

Any strength to your study? If there, add. But the main issue is outcome definition fully vaccination vs partial vaccination vs no vaccination.

Reviewer #2: INTRODUCTION/BACKGROUND

The introduction and background have failed to mention or has omitted latest data on HPV vaccine from the current statistics at MOH Uganda and recent publications in this topic. This section needs rewriting and updating recent available data to uptake and utilization of HPV vaccine in Uganda.

In 2022, WHO and UNICEF released updated data on performance of immunization post pandemic, with newer information, especially reflecting on how HPV vaccine, one of the 4 main affected vaccines are currently performing. This paper has completely ignored that data, and as such builds its case on outdated and NON-mentioned data thus doing a lot of disservice to a very important topic. The result is a poorly developed case for such an important topic and subject.

This section needs to be redone.

METHODS

The description of the conduct of the study is very brief and very shallow. What was done from the time an adolescent walked into the clinic, was offered a chance to participate, was selected… and participated to the end … this procedure has not been described. Brief mentions have been made, leaving the reviewer unable to effectively critic the sampling procedure and data collection exercise. The writers are encouraged to provide a more detailed description of how the study was conducted.

The biggest weakness of this study, that makes it fall short of a publication at the level of Plos one, is the decision of the authors to limit themselves to a quantitative study when they had a chance for a mixed methods study, through triangulation, and the unique opportunity to interview the adolescents, and invite them for focus group discussions and even conduct key informant interviews for focal health workers at the clinic and at the ministry of health. THIS constitutes a major omission and lost opportunity, that NOT ONLY weakens this study significantly, but also denies the authors an opportunity to add value to the study. This omission, has greatly weakened this study’s ability to make far reaching policy recommendations that can be adopted by the ministry of health and policy makers. This left them with a basic descriptive study, with limited experimental design use, with an attempt at logistic regression that was sop narrow as to reveal any new factors associated with low vaccine uptake and that failed to even affirm previously demonstrated factors from other similar studies in the same context. I think this failure of reproducibility of previous findings arose from a defect in the methodology and design of this study. Can this be cured or remedied and somehow make this study stronger? I do not have the answer to this.

The logistic regression, if it was done, was performed incorrectly. The factors did not yield any significant results, and even did not confirm or reject earlier study findings in this area. A posson regression would have better, but this is not possible because the factor spread and analysis frame designed by the authors was very narrow. The only way to cure this problem would have been use of triangulation, where the qualitative arm would have solved the major weaknesses and shortcomings of the quantitative arm. This was not done, thus leaving the study significantly weakened.

Why the study opted to focus on uptake and omitted aspects and drivers of completion ofHPV vaccination is not clear. Initiatives around addressing HPV vaccine failure must focus more on completion if uptake is to be of any value. This was another major omission of this study. One that at this stage may not be redeemed, yet largely undermines the utility of this study. Descriptive statistics alone at this level is not adequate.

DISCUSSION, CONCLUSSION AND RECOMMENDATIONS

The discussion is limited at narrow. This is because of several reasons:

1. The narrow approach taken in background and introduction that omitted the broad concerns and recent data around HPV vaccination globally and Uganda in particular.

2. The very narrow methodology avoiding a mixed methods approach that denied the authors the broad premise on which to make robust and cogent recommendations.

3. The failure of the authors to integrate the current post pandemic context and IA 2030 recommendations and targets into improving HPV vaccine uptake. These have completely been omitted or ignored by the authors. In the process, they have greatly weakened their own study.

Reviewer #3: This paper looks at completeness of HPV immunization and HPV knowledge in HIV+ females ages 9-19.

1. HPV vaccine is given in schools as 2 doses. HIV+ girls therefore did not receive a 3d dose in schools in Uganda. So the observations would in many ways be self-evident.

2. These are girls receiving HIV care in an HIV clinic. Why not give the vaccine in the clinic? That would solve a lot of problems.

3.Tables and Figures. Much of the same information is in the text so some of these are redundant.

4. Table 2. School not included.

5. Not sure what is meant by "cates" on line 275.

6. The authors need to put forward some solutions. A third dose in schools would stigmatize by inferring that the recipient has HIV.

7. How do results compare with HIV- girls?

**********

6. PLOS authors have the option to publish the peer review history of their article (what does this mean?). If published, this will include your full peer review and any attached files.

If you choose “no”, your identity will remain anonymous but your review may still be made public.

**Do you want your identity to be public for this peer review?** For information about this choice, including consent withdrawal, please see our Privacy Policy.

Reviewer #1: No

Reviewer #2: No

Reviewer #3: No

**********

[NOTE: If reviewer comments were submitted as an attachment file, they will be attached to this email and accessible via the submission site. Please log into your account, locate the manuscript record, and check for the action link "View Attachments". If this link does not appear, there are no attachment files.]

While revising your submission, please upload your figure files to the Preflight Analysis and Conversion Engine (PACE) digital diagnostic tool, https://pacev2.apexcovantage.com/. PACE helps ensure that figures meet PLOS requirements. To use PACE, you must first register as a user. Registration is free. Then, login and navigate to the UPLOAD tab, where you will find detailed instructions on how to use the tool. If you encounter any issues or have any questions when using PACE, please email PLOS at figures@plos.org. Please note that Supporting Information files do not need this step.

# Author comment: Author response to Decision Letter 0

**DOI:** 10.1371/journal.pone.0300155.r002

30 Sep 2023

Response letter to request for information has been attached

Attachment Submitted filename: Response letter _ draft 2-MM.docx

# Peer review: Decision Letter 1

**DOI:** 10.1371/journal.pone.0300155.r003

**Authors:** Aloysius Gonzaga Mubuuke

26 Oct 2023

PONE-D-23-17190R1UPTAKE OF HUMAN PAPILLOMA VIRUS VACCINATION AMONG ADOLESCENT GIRLS LIVING WITH HIV IN UGANDA: A CROSS SECTIONAL STUDYPLOS ONE

Dear Dr. Nakibuuka,

Thank you for submitting your manuscript to PLOS ONE. After careful consideration, we feel that it has merit but does not fully meet PLOS ONE’s publication criteria as it currently stands. Therefore, we invite you to submit a revised version of the manuscript that addresses the points raised during the review process.

==============================

The paper has improved. The authors need to present and discuss the qualitative findings as the reviewer also points this out.==============================

Please submit your revised manuscript by Dec 10 2023 11:59PM. If you will need more time than this to complete your revisions, please reply to this message or contact the journal office at plosone@plos.org. When you're ready to submit your revision, log on to https://www.editorialmanager.com/pone/ and select the 'Submissions Needing Revision' folder to locate your manuscript file.

Please include the following items when submitting your revised manuscript:

- A rebuttal letter that responds to each point raised by the academic editor and reviewer(s). You should upload this letter as a separate file labeled 'Response to Reviewers'.
- A marked-up copy of your manuscript that highlights changes made to the original version. You should upload this as a separate file labeled 'Revised Manuscript with Track Changes'.
- An unmarked version of your revised paper without tracked changes. You should upload this as a separate file labeled 'Manuscript'.

If you would like to make changes to your financial disclosure, please include your updated statement in your cover letter. Guidelines for resubmitting your figure files are available below the reviewer comments at the end of this letter.

If applicable, we recommend that you deposit your laboratory protocols in protocols.io to enhance the reproducibility of your results. Protocols.io assigns your protocol its own identifier (DOI) so that it can be cited independently in the future. For instructions see: https://journals.plos.org/plosone/s/submission-guidelines#loc-laboratory-protocols. Additionally, PLOS ONE offers an option for publishing peer-reviewed Lab Protocol articles, which describe protocols hosted on protocols.io. Read more information on sharing protocols at https://plos.org/protocols?utm_medium=editorial-email&utm_source=authorletters&utm_campaign=protocols.

We look forward to receiving your revised manuscript.

Kind regards,

Aloysius Gonzaga Mubuuke

Academic Editor

PLOS ONE

Journal Requirements:

Please review your reference list to ensure that it is complete and correct. If you have cited papers that have been retracted, please include the rationale for doing so in the manuscript text, or remove these references and replace them with relevant current references. Any changes to the reference list should be mentioned in the rebuttal letter that accompanies your revised manuscript. If you need to cite a retracted article, indicate the article’s retracted status in the References list and also include a citation and full reference for the retraction notice.

Additional Editor Comments:

The paper has greatly improved. Since there is claim that a qualitative study was conducted as a follow up, please provide the findings of the qualitative study and discuss them.

[Note: HTML markup is below. Please do not edit.]

Reviewers' comments:

Reviewer's Responses to Questions

**Comments to the Author**

1. If the authors have adequately addressed your comments raised in a previous round of review and you feel that this manuscript is now acceptable for publication, you may indicate that here to bypass the “Comments to the Author” section, enter your conflict of interest statement in the “Confidential to Editor” section, and submit your "Accept" recommendation.

Reviewer #2: All comments have been addressed

**********

2. Is the manuscript technically sound, and do the data support the conclusions?

The manuscript must describe a technically sound piece of scientific research with data that supports the conclusions. Experiments must have been conducted rigorously, with appropriate controls, replication, and sample sizes. The conclusions must be drawn appropriately based on the data presented. 

Reviewer #2: Yes

**********

3. Has the statistical analysis been performed appropriately and rigorously? 

Reviewer #2: Yes

**********

4. Have the authors made all data underlying the findings in their manuscript fully available?

The PLOS Data policy requires authors to make all data underlying the findings described in their manuscript fully available without restriction, with rare exception (please refer to the Data Availability Statement in the manuscript PDF file). The data should be provided as part of the manuscript or its supporting information, or deposited to a public repository. For example, in addition to summary statistics, the data points behind means, medians and variance measures should be available. If there are restrictions on publicly sharing data—e.g. participant privacy or use of data from a third party—those must be specified.

Reviewer #2: No

**********

5. Is the manuscript presented in an intelligible fashion and written in standard English?

PLOS ONE does not copyedit accepted manuscripts, so the language in submitted articles must be clear, correct, and unambiguous. Any typographical or grammatical errors should be corrected at revision, so please note any specific errors here.

Reviewer #2: Yes

**********

6. Review Comments to the Author

Please use the space provided to explain your answers to the questions above. You may also include additional comments for the author, including concerns about dual publication, research ethics, or publication ethics. (Please upload your review as an attachment if it exceeds 20,000 characters)

Reviewer #2: The authors in their response to the question on methodology have stated: Concerning the missed mixed methods approach. We indeed did a follow up study which was a qualitative study to explain the results from the quantitative study. However, the qualitative study findings would overshadow the key findings in this quantitative study.

My question: What were the findings of the qualitative study? Can they provide a summary that discusses these findings? If the findings cannot be provided here, what is the reason for the redaction of those findings? Can they at the very least be discussed in the discussion section? Is there a reason why they are being deliberately omitted? Could it be that they were not covered within the expanse of the ethical approve for this work and so falls under a remit that hinders their introduction?

**********

7. PLOS authors have the option to publish the peer review history of their article (what does this mean?). If published, this will include your full peer review and any attached files.

If you choose “no”, your identity will remain anonymous but your review may still be made public.

**Do you want your identity to be public for this peer review?** For information about this choice, including consent withdrawal, please see our Privacy Policy.

Reviewer #2: No

**********

[NOTE: If reviewer comments were submitted as an attachment file, they will be attached to this email and accessible via the submission site. Please log into your account, locate the manuscript record, and check for the action link "View Attachments". If this link does not appear, there are no attachment files.]

While revising your submission, please upload your figure files to the Preflight Analysis and Conversion Engine (PACE) digital diagnostic tool, https://pacev2.apexcovantage.com/. PACE helps ensure that figures meet PLOS requirements. To use PACE, you must first register as a user. Registration is free. Then, login and navigate to the UPLOAD tab, where you will find detailed instructions on how to use the tool. If you encounter any issues or have any questions when using PACE, please email PLOS at figures@plos.org. Please note that Supporting Information files do not need this step.

# Author comment: Author response to Decision Letter 1

**DOI:** 10.1371/journal.pone.0300155.r004

18 Jan 2024

Responses uploaded under the attachment section

# Editor report: Decision Letter 2

**DOI:** 10.1371/journal.pone.0300155.r005

**Authors:** Aloysius Gonzaga Mubuuke

19 Jan 2024

PONE-D-23-17190R2UPTAKE OF HUMAN PAPILLOMA VIRUS VACCINATION AMONG ADOLESCENT GIRLS LIVING WITH HIV IN UGANDA: A CROSS SECTIONAL STUDYPLOS ONE

Dear Dr. Nakibuuka,

Thank you for submitting your manuscript to PLOS ONE. After careful consideration, we feel that it has merit but does not fully meet PLOS ONE’s publication criteria as it currently stands. Therefore, we invite you to submit a revised version of the manuscript that addresses the points raised during the review process.

==============================

**ACADEMIC EDITOR:**

The revised paper is indeed very strong. The qualitative findings included do provide more rigor to the study. Some minor observation that should be addressed by the authors:

In the results section of the Qualitative findings, Line 335, the naming of the themes should be revised. Currently, the three themes are named with very long sentences that read like just descriptions. The name of a theme should be shorter and concise and the description of the theme can come just after the name. I offer some suggestions below on how to name your three themes:

Theme 1: Limited information about HPV Vaccination

Theme 2: Parental influence on adolescent decisions

Theme 3: Inadequacy of HPV Vaccination services

==============================

Please submit your revised manuscript by Mar 04 2024 11:59PM. If you will need more time than this to complete your revisions, please reply to this message or contact the journal office at plosone@plos.org. When you're ready to submit your revision, log on to https://www.editorialmanager.com/pone/ and select the 'Submissions Needing Revision' folder to locate your manuscript file.

Please include the following items when submitting your revised manuscript:

- A rebuttal letter that responds to each point raised by the academic editor and reviewer(s). You should upload this letter as a separate file labeled 'Response to Reviewers'.
- A marked-up copy of your manuscript that highlights changes made to the original version. You should upload this as a separate file labeled 'Revised Manuscript with Track Changes'.
- An unmarked version of your revised paper without tracked changes. You should upload this as a separate file labeled 'Manuscript'.

If you would like to make changes to your financial disclosure, please include your updated statement in your cover letter. Guidelines for resubmitting your figure files are available below the reviewer comments at the end of this letter.

If applicable, we recommend that you deposit your laboratory protocols in protocols.io to enhance the reproducibility of your results. Protocols.io assigns your protocol its own identifier (DOI) so that it can be cited independently in the future. For instructions see: https://journals.plos.org/plosone/s/submission-guidelines#loc-laboratory-protocols. Additionally, PLOS ONE offers an option for publishing peer-reviewed Lab Protocol articles, which describe protocols hosted on protocols.io. Read more information on sharing protocols at https://plos.org/protocols?utm_medium=editorial-email&utm_source=authorletters&utm_campaign=protocols.

We look forward to receiving your revised manuscript.

Kind regards,

Aloysius Gonzaga Mubuuke

Academic Editor

PLOS ONE

Journal Requirements:

Please review your reference list to ensure that it is complete and correct. If you have cited papers that have been retracted, please include the rationale for doing so in the manuscript text, or remove these references and replace them with relevant current references. Any changes to the reference list should be mentioned in the rebuttal letter that accompanies your revised manuscript. If you need to cite a retracted article, indicate the article’s retracted status in the References list and also include a citation and full reference for the retraction notice.

Additional Editor Comments:

The revised paper is indeed very strong. The qualitative findings included do provide more rigor to the study. Some minor observation that should be addressed by the authors:

In the results section of the Qualitative findings, Line 335, the naming of the themes should be revised. Currently, the three themes are named with very long sentences that read like just descriptions. The name of a theme should be shorter and concise and the description of the theme can come just after the name. I offer some suggestions below on how to name your three themes:

Theme 1: Limited information about HPV Vaccination

Theme 2: Parental influence on adolescent decisions

Theme 3: Inadequacy of HPV Vaccination services

[Note: HTML markup is below. Please do not edit.]

[NOTE: If reviewer comments were submitted as an attachment file, they will be attached to this email and accessible via the submission site. Please log into your account, locate the manuscript record, and check for the action link "View Attachments". If this link does not appear, there are no attachment files.]

While revising your submission, please upload your figure files to the Preflight Analysis and Conversion Engine (PACE) digital diagnostic tool, https://pacev2.apexcovantage.com/. PACE helps ensure that figures meet PLOS requirements. To use PACE, you must first register as a user. Registration is free. Then, login and navigate to the UPLOAD tab, where you will find detailed instructions on how to use the tool. If you encounter any issues or have any questions when using PACE, please email PLOS at figures@plos.org. Please note that Supporting Information files do not need this step.

# Author comment: Author response to Decision Letter 2

**DOI:** 10.1371/journal.pone.0300155.r006

22 Jan 2024

Response letter to respond to reviewer comments has been attached

Attachment Submitted filename: Response to Reviewers 22.01.docx

# Editor report: Decision Letter 3

**DOI:** 10.1371/journal.pone.0300155.r007

**Authors:** Aloysius Gonzaga Mubuuke

30 Jan 2024

PONE-D-23-17190R3Uptake of Human Papilloma Virus Vaccination Among Adolescent Girls Living with HIV In Uganda: A mixed method StudyPLOS ONE

Dear Dr. Nakibuuka,

Thank you for submitting your manuscript to PLOS ONE. After careful consideration, we feel that it has merit but does not fully meet PLOS ONE’s publication criteria as it currently stands. Therefore, we invite you to submit a revised version of the manuscript that addresses the points raised during the review process.

 Please submit your revised manuscript by Mar 15 2024 11:59PM. If you will need more time than this to complete your revisions, please reply to this message or contact the journal office at plosone@plos.org. When you're ready to submit your revision, log on to https://www.editorialmanager.com/pone/ and select the 'Submissions Needing Revision' folder to locate your manuscript file.

Please include the following items when submitting your revised manuscript:

- A rebuttal letter that responds to each point raised by the academic editor and reviewer(s). You should upload this letter as a separate file labeled 'Response to Reviewers'.
- A marked-up copy of your manuscript that highlights changes made to the original version. You should upload this as a separate file labeled 'Revised Manuscript with Track Changes'.
- An unmarked version of your revised paper without tracked changes. You should upload this as a separate file labeled 'Manuscript'.

If you would like to make changes to your financial disclosure, please include your updated statement in your cover letter. Guidelines for resubmitting your figure files are available below the reviewer comments at the end of this letter.

If applicable, we recommend that you deposit your laboratory protocols in protocols.io to enhance the reproducibility of your results. Protocols.io assigns your protocol its own identifier (DOI) so that it can be cited independently in the future. For instructions see: https://journals.plos.org/plosone/s/submission-guidelines#loc-laboratory-protocols. Additionally, PLOS ONE offers an option for publishing peer-reviewed Lab Protocol articles, which describe protocols hosted on protocols.io. Read more information on sharing protocols at https://plos.org/protocols?utm_medium=editorial-email&utm_source=authorletters&utm_campaign=protocols.

We look forward to receiving your revised manuscript.

Kind regards,

Aloysius Gonzaga Mubuuke

Academic Editor

PLOS ONE

Journal Requirements:

Please review your reference list to ensure that it is complete and correct. If you have cited papers that have been retracted, please include the rationale for doing so in the manuscript text, or remove these references and replace them with relevant current references. Any changes to the reference list should be mentioned in the rebuttal letter that accompanies your revised manuscript. If you need to cite a retracted article, indicate the article’s retracted status in the References list and also include a citation and full reference for the retraction notice.

**Additional Editor Comments:**==============================

Some minor comments still to address in relation to your themes in the qualitative part. While the authors have edited the naming of the qualitative themes while introducing them (Lines 339-342), the new names have not been adopted in the subsequent description of the themes. For example, while the name for theme 1 has been changed in 339, the old name still remains in line 348-349. Change the naming of the themes even before the participant quotations.==============================

[Note: HTML markup is below. Please do not edit.]

[NOTE: If reviewer comments were submitted as an attachment file, they will be attached to this email and accessible via the submission site. Please log into your account, locate the manuscript record, and check for the action link "View Attachments". If this link does not appear, there are no attachment files.]

While revising your submission, please upload your figure files to the Preflight Analysis and Conversion Engine (PACE) digital diagnostic tool, https://pacev2.apexcovantage.com/. PACE helps ensure that figures meet PLOS requirements. To use PACE, you must first register as a user. Registration is free. Then, login and navigate to the UPLOAD tab, where you will find detailed instructions on how to use the tool. If you encounter any issues or have any questions when using PACE, please email PLOS at figures@plos.org. Please note that Supporting Information files do not need this step.

# Author comment: Author response to Decision Letter 3

**DOI:** 10.1371/journal.pone.0300155.r008

15 Feb 2024

Responded in the comments

Attachment Submitted filename: Response to Reviewers 22.01.docx

# Editor report: Decision Letter 4

**DOI:** 10.1371/journal.pone.0300155.r009

**Authors:** Aloysius Gonzaga Mubuuke

22 Feb 2024

Uptake of Human Papilloma Virus Vaccination Among Adolescent Girls Living with HIV In Uganda: A mixed method Study

PONE-D-23-17190R4

Dear Dr. Nakibuuka,

We’re pleased to inform you that your manuscript has been judged scientifically suitable for publication and will be formally accepted for publication once it meets all outstanding technical requirements.

Within one week, you’ll receive an e-mail detailing the required amendments. When these have been addressed, you’ll receive a formal acceptance letter and your manuscript will be scheduled for publication.

An invoice for payment will follow shortly after the formal acceptance. To ensure an efficient process, please log into Editorial Manager at http://www.editorialmanager.com/pone/, click the 'Update My Information' link at the top of the page, and double check that your user information is up-to-date. If you have any billing related questions, please contact our Author Billing department directly at authorbilling@plos.org.

If your institution or institutions have a press office, please notify them about your upcoming paper to help maximize its impact. If they’ll be preparing press materials, please inform our press team as soon as possible -- no later than 48 hours after receiving the formal acceptance. Your manuscript will remain under strict press embargo until 2 pm Eastern Time on the date of publication. For more information, please contact onepress@plos.org.

Kind regards,

Aloysius Gonzaga Mubuuke

Academic Editor

PLOS ONE

Additional Editor Comments (optional):

Reviewers' comments:

# Editor report: Acceptance letter

**DOI:** 10.1371/journal.pone.0300155.r010

**Authors:** Aloysius Gonzaga Mubuuke

26 Apr 2024

PONE-D-23-17190R4 

PLOS ONE

Dear Dr. Nakibuuka, 

I'm pleased to inform you that your manuscript has been deemed suitable for publication in PLOS ONE. Congratulations! Your manuscript is now being handed over to our production team.

At this stage, our production department will prepare your paper for publication. This includes ensuring the following:

* All references, tables, and figures are properly cited

* All relevant supporting information is included in the manuscript submission,

* There are no issues that prevent the paper from being properly typeset

If revisions are needed, the production department will contact you directly to resolve them. If no revisions are needed, you will receive an email when the publication date has been set. At this time, we do not offer pre-publication proofs to authors during production of the accepted work. Please keep in mind that we are working through a large volume of accepted articles, so please give us a few weeks to review your paper and let you know the next and final steps. 

Lastly, if your institution or institutions have a press office, please let them know about your upcoming paper now to help maximize its impact. If they'll be preparing press materials, please inform our press team within the next 48 hours. Your manuscript will remain under strict press embargo until 2 pm Eastern Time on the date of publication. For more information, please contact onepress@plos.org.

If we can help with anything else, please email us at customercare@plos.org.

Thank you for submitting your work to PLOS ONE and supporting open access. 

Kind regards, 

PLOS ONE Editorial Office Staff

on behalf of

Dr. Aloysius Gonzaga Mubuuke 

Academic Editor

PLOS ONE
